# Tandem Administration of Prostaglandin F_2α_ and Gonadotropin-Releasing Hormone in Beef Heifers and Cows as a Convergent Presynchronization Method in the 7 & 7 Synch Protocol

**DOI:** 10.3390/ani15091329

**Published:** 2025-05-05

**Authors:** Lucas J. Palcheff, Genevieve M. VanWye, Kimberly R. Ricardo, Kendal L. Green, Franklin J. Even, Samantha R. Roberts, Adella B. Lonas, Christine M. Spinka, Scott E. Poock, Saulo Menegatti Zoca, Jessica N. Drum, Jordan M. Thomas

**Affiliations:** 1 Division of Animal Sciences, University of Missouri, Columbia, MO 65211, USA; 2 Department of Animal Science, South Dakota State University, Brookings, SD 57007, USA; 3 Department of Animal Science, University of Tennessee, Knoxville, TN 37996, USA; 4 School of Medicine, University of Missouri, Columbia, MO 65211, USA; 5 College of Veterinary Medicine, University of Missouri, Columbia, MO 65211, USA

**Keywords:** anestrus, acyclic, follicular dynamics, ovulatory stimulus

## Abstract

An experiment was designed to evaluate modifications to the 7 & 7 Synch protocol, with the hypothesis that tandem administration of prostaglandin F2α (PG) and gonadotropin-releasing hormone (GnRH) during the presynchronization portion of the protocol would generate a convergent ovarian response among anestrous and estrous cycling females. Nulliparous, primiparous, and multiparous Angus and Angus-cross females were blocked based on parity and pre-treatment luteal status and, within a block, randomly assigned to one of three treatments. The females assigned to the 7 & 7 Synch treatment (n = 765) received an intravaginal progesterone-releasing insert (CIDR^®^) and an administration of PG on Day 0, GnRH on Day 7, and PG concurrent with CIDR removal on Day 14. Estrotect™ estrus detection aids were applied on Day 14 at the time of CIDR removal and PG administration. The females assigned to the 7 & 7 + G treatment (n = 769) received the same treatment schedule, with the modification that GnRH was administered in tandem with PG on Day 0. The females assigned to the 7 & 7 + G + P treatment (n = 786) received the same treatment schedule, with the modification that GnRH was administered in tandem with PG on both Days 0 and 7. Fixed-time artificial insemination (FTAI) was performed at 54 ± 2 h (nulliparous) or 66 ± 2 h (primiparous and multiparous) after CIDR removal. Transrectal ovarian ultrasonography was performed on Days 7 and 14 to characterize the largest follicle diameter (LFD) and the presence of corpora lutea (CL). Estrus prior to FTAI was characterized using estrus detection aids. Pregnancy status was determined via transrectal ultrasonography approximately 60–100 days after FTAI. The proportion of multiparous females with CL on Day 14 tended to be greater following treatment with 7 & 7 Synch or 7 & 7 + G (90% and 87%, respectively), as compared to females treated with 7 & 7 + G + P (82%; *p* = 0.09). A greater proportion of multiparous females expressed behavioral estrus prior to FTAI when treated with 7 & 7 + G + P (90%) as compared to multiparous females treated with 7 & 7 + G (79%; *p* < 0.002) or 7 & 7 Synch (78%; *p* = 0.0004); however, pregnancy rates resulting from FTAI did not differ based on treatment. In summary, tandem administration of GnRH and PG on Days 0 and 7 of the 7 & 7 Synch protocol effectively generated a convergent ovarian response among females with variable pretreatment luteal status but failed to enhance pregnancy rates to FTAI.

## Simple Summary

In the cow–calf industry, estrus synchronization and artificial insemination are reproductive technologies that improve conception rates early in the breeding season. These technologies concentrate calving earlier in the season, increasing calf weaning weights and enhancing producer profitability. A recently developed estrus synchronization protocol, 7 & 7 Synch, has demonstrated improvements in pregnancy rates resulting from artificial insemination. However, improvements in pregnancy results using 7 & 7 Synch may be limited to cyclic females. Acyclic females (i.e., females not already undergoing regular estrous cycles prior to the initiation of the synchronization protocol) lack a functional corpus luteum, rendering them unresponsive to the prostaglandin F2α administered at the initiation of 7 & 7 Synch. Commercial cow–calf herds often consist of a mix of acyclic and cycling females, posing a challenge for successful estrus synchronization. Thus, differences in response of cyclic versus acyclic females to an estrus synchronization protocol limits overall protocol efficacy and negatively impacts producer profitability. This study aimed to evaluate a presynchronization strategy intended to enhance synchronization success in acyclic females while maintaining the results achieved in cyclic females using 7 & 7 Synch. Incorporating additional hormones during presynchronization facilitated a convergence in ovarian presentation among females with varying pretreatment estrous cyclicity status; however, it failed to improved P/AI.

## 1. Introduction

A significant challenge with estrus synchronization (ES) protocols designed for beef cattle, such as the 7-day CO-Synch + CIDR, is the inconsistent response to gonadotropin-releasing hormone (GnRH) administered at protocol initiation to induce a new follicular wave. When administered at a random point of the estrous cycle, GnRH induces ovulation in approximately 66% of cows [1]. The failure of some females to ovulate results in subsets of individuals with divergent ovarian presentations, leading to greater variability in the timing of estrus and ovulation, potentially reducing the pregnancy rate to fixed-time artificial insemination (P/AI). Presynchronization strategies aim to manage the stage of cycle prior to GnRH and, thus, increase the proportion of females responding to GnRH [2]. However, in the beef industry, protocols with several additional animal handling events (i.e., presynchronization strategies like Double Ovsynch, used in dairy cattle) are less practical.

The 7 & 7 Synch protocol introduces two presynchronization components to the 7-day CO-Synch + CIDR protocol in a single handling event, as follows: an administration of prostaglandin F2α (PG) and treatment with an intravaginal progesterone-releasing device (CIDR) prior to the administration of GnRH. Administering PG prior to GnRH can increase the proportion of females presenting with a GnRH-responsive follicle [3]. In the absence of a corpus luteum (CL), subluteal progesterone (P4) concentrations from the CIDR allow for a pulsatile pattern of luteinizing hormone (LH) release with adequate frequency to stimulate follicular growth and support continued dominance [4,5]. Compared to the 7-day CO-Synch + CIDR protocol, the 7 & 7 Synch protocol increased the largest follicle diameter (LFD) at GnRH administration, reduced variation in luteal status on Day 14 [6,7], increased the proportion of females expressing behavioral estrus, and improved P/AI in some [7,8,9], but not all [10,11], studies. Differing observations related to P/AI raises questions, particularly with respect to the influence of cyclicity status and ovarian presentation at the start of treatment.

The administration of PG is only effective in inducing luteolysis in cycling females between Days 6 and 16 of the estrous cycle [12]; therefore, females on Days 1 to 5 of the estrous cycle at the start of the 7 & 7 Synch protocol are likely to maintain CL and, rather than forming a persistent follicle, have a variable LFD when GnRH is administered 7 days later. Acyclic females (i.e., anestrous cows and peripubertal heifers) of course lack CL and, thus, also do not undergo luteolysis in response to PG. Variance among females in ovarian response is a source of potential variance in the timing of estrus and ovulation, which can affect P/AI when FTAI is performed.

Modern protocols used to facilitate FTAI have a reasonable degree of efficacy across females of varying cyclicity status [1,13]. However, the objective of this experiment was to achieve more uniform control over luteal and follicular dynamics and improve the proportion of acyclic females conceiving to FTAI. Incorporating GnRH at presynchronization initiation can induce ovulation in a proportion of acyclic females [14,15,16], forming CL and initiating a new synchronized follicular wave. Therefore, we hypothesized that the tandem administration of GnRH and PG on Days 0 and 7 of the 7 & 7 Synch protocol would reduce variation in luteal and follicular status on Day 14, increase the proportion of females expressing behavioral estrus prior to FTAI, and improve P/AI.

## 2. Materials and Methods

### 2.1. Animals and Estrus Synchronization

**Figure 1:**
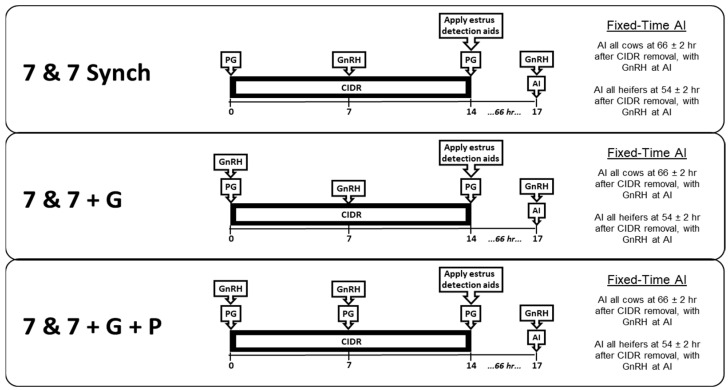
Treatment schedule for beef females enrolled in a fixed-time AI protocol with presynchronization. Females assigned to the 7 & 7 Synch treatment (n = 765) received an intravaginal progesterone-releasing insert (CIDR^®^) and administration of prostaglandin F2α (PG) on Day 0, administration of gonadotropin-releasing hormone (GnRH) on Day 7, and administration of PG concurrent with CIDR removal on Day 14. Estrotect™ estrus detection aids were applied on Day 14 at the time of CIDR removal and PG administration. Females assigned to the 7 & 7 + G treatment (n = 769) received the same treatment schedule as females on the 7 & 7 Synch treatment, with the modification that GnRH was administered in tandem with PG on Day 0. Females assigned to the 7 & 7 + G + P treatment (n = 786) received the same treatment schedule as females on the 7 & 7 Synch treatment, with the modification that GnRH was administered in tandem with PG on both Day 0 and Day 7 of the treatment schedule. Fixed-time artificial insemination (FTAI) was performed at 54 ± 2 h after CIDR removal for nulliparous females and at 66 ± 2 h for primiparous and multiparous females. Coincident with FTAI, all females were administered GnRH. Expression of behavioral estrus prior to FTAI was recorded at the time of FTAI based on Estrotect™ activation score.

Beginning in the spring breeding season of 2022 and ending in the fall of 2023, estrus was synchronized in 2320 beef females (n = 575 nulliparous; n = 338 primiparous; n = 1407 multiparous) across locations (n = 13) in Missouri, South Dakota, and Tennessee (Table 1). Pre-treatment ovarian status was assessed on Day 0 via transrectal ovarian ultrasonography, with an assessment of luteal status, after which females without CL were classified as non-luteal, and females with CL were classified as luteal. Within each location, females were blocked based on parity and pre-treatment luteal status and were randomly assigned, within a block, to one of three treatments (Figure 1). The number of females by parity and treatment, as well as their pre-treatment luteal status classification by treatment and parity, is summarized in Table 2. Pre-treatment luteal status did not differ across treatments.

Females assigned to the 7 & 7 Synch treatment received a 1.38 g CIDR (EAZI-Breed CIDR^®^ Zoetis, Madison, NJ, USA) coincident with the administration of PG (500 µg cloprostenol sodium i.m.; Estrumate; Merck Animal Health, Madison, NJ; or 25 mg dinoprost tromethamine i.m.; Lutalyse; Zoetis, Madison, NJ; consistent across treatments within the location) on Day 0, administration of GnRH (100 µg gonadorelin acetate i.m.; Fertagyl; Merck Animal Health, Madison, NJ; or 100 µg gonadorelin hydrochloride i.m.; Factrel; Zoetis, Madison, NJ; consistent across treatments within the location) on Day 7, and administration of PG with CIDR removal on Day 14. Concurrent with PG administration and CIDR removal, all females had an estrus detection patch (Estrotect; Rockway Inc, Spring Valley, WI, USA) placed on their tail-head. At FTAI on Day 17, all females were administered GnRH. Females assigned to the 7 & 7 + G treatment received the same treatment schedule as females treated with 7 & 7 Synch, with the modification that GnRH was administered in tandem with PG on Day 0. Females assigned to the 7 & 7 + G + P treatment received the same treatment schedule as females treated with 7 & 7 Synch, with the modification that GnRH was administered in tandem with PG on both Day 0 and Day 7 of the treatment schedule. Females were exposed to bulls for natural service beginning 14 days after FTAI.

### 2.2. Ovarian Ultrasonography

Transrectal ovarian ultrasonography was performed on Days 7 and 14 using a Sonosite EDGE equipped with an L52 10.0–5.0 MHz linear-array transducer (SonoSite Inc., Bothwell, WA, USA) in locations 3, 4, 6, 10, 11, and 13, and an Ibex EVO II equipped with a 2.0–14.0 MHz linear-array transducer (Ibex, Washington, DC, USA) in locations 7, 8, 9, and 12. Transrectal ovarian ultrasonography was used to determine the LFD measurement and presence and number of CL. The LFD was determined based on the average height and width of the follicular measurement, as assessed via a caliper tool in each ultrasound machine. The CL status was determined by observing one or more distinct luteal structures, with the number of CL noted when more than one was present.

On Days 7 and 14, CL status was recorded for all females in locations 6, 8, and 12. When time and labor were constrained, transrectal ovarian ultrasonography was performed on Days 7 and 14 to determine CL status on a representative subset of females from each treatment. This subset included locations 3, 4, 7, 9, 10, 11, and 13 for Day 7 CL status, and locations 3, 7, 9, 10, 11, and 13 for Day 14 CL status. A measurement of LFD was collected for all females in locations 6 and 12 on Days 7 and 14; and on a representative subset in locations 3, 4, and 10–13 on Day 7 and in locations 3 and 10–13 on Day 14.

### 2.3. Estrus Detection and Artificial Insemination

Estrus detection aids (Estrotect, Rockway Inc, Spring Valley, WI, USA) were applied to all females on Day 14 at the time of final PG administration and CIDR removal and secured using a spray adhesive. Estrus detection aid (patch) activation was recorded at the time of FTAI. Patch “activation”, or removal of the coating from the Estrotect patch, was scored on a scale of 0 to 4 (0 = missing patch; 1 = 0–25% of patch activated; 2 = 25–50% of patch activated; 3 = 50–75% of patch activated; 4 = 75–100% of patch activated) [17]. Females were classified as having expressed behavioral estrus if 50% or more of the patch was activated (score = 3 or 4) or if the patch was missing (score = 0). Females were classified as non-estrous if less than 50% of their patch was activated (score = 1 or 2).

On Day 17, FTAI was performed at 54 ± 2 h after PG administration for nulliparous females and at 66 ± 2 h after PG administration for primiparous and multiparous females. Females were inseminated with either conventional (n = 2083) or sex-sorted (n = 236) semen, with semen type being balanced across treatments within each location. Multiple technicians performed insemination, with technicians being balanced by location, treatment, and semen type.

### 2.4. Pregnancy Diagnosis

Pregnancy rate to AI was determined 60 to 100 days after FTAI with transrectal ultrasonography, using a SonoSite EDGE equipped with an L52 10.0–5.0 MHz linear-array transducer (SonoSite Inc., Bothwell, WA, USA) in locations 1–6, 10, 11, and 13; and with an Ibex EVO II equipped with a 2.0–14.0 MHz linear-array transducer (Ibex, Washington, DC, USA) in locations 7–9 and 12. Experienced diagnosticians evaluated fetal size measurements and development to accurately distinguish pregnancies resulting from FTAI and those from subsequent natural service, which was made possible by the lack of natural service bull exposure for two weeks after FTAI.

### 2.5. Statistical Analysis

Statistical analyses were performed using a Statistical Analysis System (SAS 9.4 Inst. Inc., Cary, NC, USA). Mixed models (GLIMMIX procedure of SAS) using the binomial distribution link logit function were used to analyze CL status on Day 7 and Day 14, the proportion of females expressing behavioral estrus prior to FTAI, and P/AI. A mixed model (GLIMMIX procedure of SAS) was used to analyze the continuous variable response of LFD. Location was included as a random effect in all models.

For the analysis of Day 7 CL status and Day 7 LFD, the fixed effects included in these models were treatment, pre-treatment luteal status, and parity. All two-way and three-way interactions were tested for inclusion in these models, and non-significant interaction effects (*p* > 0.10) were eliminated from the model in a backwards elimination process. When appropriate for tests of certain hypotheses, the 7 & 7 Synch treatment was compared to the pooled treatments of 7 & 7 + G and 7 & 7 + G + P, as they were identical until Day 7 of the treatment schedule. For the analysis of Day 14 LFD, the fixed effects included in the model were treatment, pre-treatment luteal status, and Day 14 CL status. All two-way and three-way interactions were included in this model. For the analysis of Day 14 CL status and estrus, the fixed effects included in these models were treatment, pre-treatment luteal status, and parity. All two-way and three-way interactions were included in these models. For the analysis of P/AI, the fixed effects included in the model were estrus, treatment, Day 14 CL status, parity, and semen type. All two-way and three-way interactions were tested for potential inclusion in the model, and non-significant interaction effects (*p* > 0.10) involving parity and semen type were eliminated from the model in a backwards elimination process. As a result, the final model for the analysis of P/AI included all two-way and three-way interactions for the fixed effects of estrus, treatment, and Day 14 CL status.

## 3. Results

### 3.1. Largest Follicle Diameter

Day 7 LFD was affected by pre-treatment luteal status (*p* = 0.0005), parity (*p* < 0.0001), and the interaction of treatment and parity (*p* = 0.01). The two-way interactions of pre-treatment luteal status and parity, pre-treatment luteal status and treatment, as well as the three-way interaction of treatment, parity, and pre-treatment luteal status, did not influence Day 7 LFD (*p* = 0.94, *p* = 0.16, *p* = 0.55, respectively). Day 7 LFD measurements, recorded via transrectal ovarian ultrasonography, are summarized by treatment and parity in Table 3, and by treatment and pre-treatment luteal status in Table 4. Females classified as luteal on Day 0 had a larger (*p* = 0.0005) Day 7 LFD (12.2 ± 0.17 mm) compared to females that were non-luteal on Day 0 (11.2 ± 0.21 mm). Additionally, Day 7 LFD was larger (*p* < 0.0001) among multiparous (12.6 ± 0.23 mm) and primiparous (11.8 ± 0.39 mm) females compared to nulliparous females (10.6 ± 0.26 mm). Multiparous females treated with 7 & 7 Synch presented with a larger (*p* < 0.01) Day 7 LFD (13.1 ± 0.29 mm) compared to females treated with the modified treatments 7 & 7 + G (12.2 ± 0.20 mm) or 7 & 7 + G + P (12.4 ± 0.20 mm). Treatment did not affect Day 7 LFD in primiparous (*p* = 0.39) or nulliparous (*p* = 0.44) females.

Day 14 LFD tended (*p* = 0.08) to be influenced by the interaction of pre-treatment luteal status and Day 14 CL status. Pre-treatment luteal status and Day 14 CL status did not impact Day 14 LFD (*p* = 0.50 and *p* = 0.43, respectively). The two-way interactions of pre-treatment luteal status and treatment, treatment and Day 14 CL status, as well as the three-way interaction of pre-treatment luteal status, treatment, and Day 14 CL status, did not affect Day 14 LFD (*p* = 0.60, *p* = 0.22, *p* = 0.29, respectively). Day 14 LFD is summarized by treatment and parity in Table 3, and by treatment and pre-treatment luteal status in Table 4. Females classified as luteal on Day 0 and without CL on Day 14 presented with a larger (*p* = 0.03) LFD on Day 14 (11.7 ± 0.40 mm) when compared to females that were non-luteal on Day 0 and with CL on Day 14 (11.1 ± 0.36 mm). In addition, females classified as luteal on Day 0 and without CL on Day 14 had a larger (*p* = 0.03) LFD on Day 14 (11.7 ± 0.40 mm) when compared to females that were luteal on Day 0 and presenting with multiple CL on Day 14 (10.0 ± 0.56 mm). Day 14 LFD was not further impacted by the interaction of pre-treatment luteal status and Day 14 CL status (*p* > 0.05).

### 3.2. Corpora Lutea Status

Day 7 CL status was affected by treatment (*p* < 0.0001) and parity (*p* < 0.0001). Pre-treatment luteal status did not influence Day 7 CL status (*p* = 0.27). The two-way interactions of pre-treatment luteal status and parity, pre-treatment luteal status and treatment, treatment and parity, as well as the three-way interaction of treatment, parity, and pre-treatment luteal status, did not affect Day 7 CL status (*p* = 0.79, *p* = 0.75, *p* = 0.13, *p* = 0.37, respectively). Day 7 CL status, as assessed via transrectal ovarian ultrasonography, is summarized by treatment and parity in Table 3, and by treatment and pre-treatment luteal status in Table 4. A greater proportion (*p* < 0.0001) of females presented with CL on Day 7 when treated with the modified treatments 7 & 7 + G or 7 & 7 + G + P (63%) when compared to females treated with 7 & 7 Synch (38%). Additionally, a greater proportion (*p* < 0.0001) of females were observed with CL on Day 7 if primiparous (71%) or multiparous (68%) compared to those that were nulliparous (38%).

Day 14 CL status was affected by parity (*p* < 0.0001) and tended to be influenced by pre-treatment luteal status (*p* = 0.06) and the interaction of treatment and parity (*p* = 0.09). The two-way interactions of pre-treatment luteal status and parity, pre-treatment luteal status and treatment, as well as the three-way interaction of treatment, parity, and pre-treatment luteal status did not affect Day 14 CL status (*p* = 0.82, *p* = 0.17, *p* = 0.47, respectively). Day 14 CL status, as assessed via transrectal ovarian ultrasonography, is summarized by treatment and parity in Table 3, and by treatment and pre-treatment luteal status in Table 4. Pre-treatment luteal status tended to positively influence Day 14 CL status (*p* = 0.06), with a tendency for a greater proportion of females to have CL on Day 14 if having also presented as luteal on Day 0 (80%), as compared to those that were non-luteal on Day 0 (68%). Additionally, Day 14 CL status increased by ascending parity (*p* < 0.0001), with a greater proportion of multiparous (86%) and primiparous (71%) females presenting with CL on Day 14, as compared to nulliparous females (56%). Lastly, the proportion of multiparous females with CL on Day 14 tended to be greater following treatment with 7 & 7 Synch or 7 & 7 + G (90% and 87%, respectively), as compared to females treated with 7 & 7 + G + P (82%; *p* = 0.09). There was a tendency (*p* = 0.08) for a greater proportion of primiparous females treated with 7 & 7 Synch to present with CL on Day 14 when compared to primiparous females treated with 7 & 7 + G (74% vs. 69%, respectively), whereas, in nulliparous females, Day 14 CL status was not impacted by treatment (*p* > 0.30).

### 3.3. Expression of Behavioral Estrus

The proportion of females expressing behavioral estrus prior to FTAI was affected by pre-treatment luteal status (*p* < 0.0001) and the interaction of treatment and parity (*p* = 0.002). The two-way interactions of pre-treatment luteal status and parity, pre-treatment luteal status and treatment, as well as the three-way interaction of treatment, parity, and pre-treatment luteal status, did not influence estrus (*p* = 0.35, *p* = 0.60, *p* = 0.55, respectively). The proportion of females expressing behavioral estrus prior to FTAI is summarized by treatment and parity in Table 5. A greater proportion (*p* < 0.0001) of females classified as luteal on Day 0 expressed behavioral estrus prior to FTAI (84%) when compared to non-luteal females (64%). A greater proportion of multiparous females expressed behavioral estrus prior to FTAI when treated with 7 & 7 + G + P (90%), as compared to multiparous females treated with 7 & 7 + G (79%; *p* < 0.002) or 7 & 7 Synch (78%; *p* = 0.0004). In nulliparous females, estrus prior to FTAI was not influenced by treatment (*p* = 0.37).

### 3.4. Pregnancy Rate to Fixed-Time Artificial Insemination

Pregnancy rate to FTAI was affected by the interaction of estrus and Day 14 CL status (*p* < 0.03) and tended (*p* = 0.06) to be influenced by the interaction of treatment and Day 14 CL status. Treatment, parity, and semen type did not impact P/AI (*p* = 0.20, *p* = 0.13, *p* = 0.52, respectively). The two-way interaction between estrus and treatment, as well as the three-way interaction of treatment, estrus, and Day 14 CL status, did not affect P/AI (*p* = 0.29 and *p* = 0.79, respectively). Pregnancy rate to FTAI is summarized by treatment and Day 14 CL status in Table 6. Females that had no CL on Day 14 after treatment with 7 & 7 + G tended to have poorer pregnancy rates to FTAI (43%; *p* = 0.06) compared to females across treatments that had CL on Day 14. Greater P/AI was observed among females expressing behavioral estrus prior to FTAI compared to females failing to express behavioral estrus (59% vs. 35%; *p* < 0.0001), but there was further separation within estrous and non-estrous animals due to the interaction with Day 14 CL status. Among females that expressed behavioral estrus prior to FTAI, greater P/AI was observed among females that had CL on Day 14 (62%) compared to those without CL on Day 14 (56%; *p* < 0.03). Females that failed to express behavioral estrus prior to FTAI and that did not present with CL on Day 14 had reduced P/AI (28%; *p* < 0.005) compared to all other groups within the estrus and Day 14 CL status interaction model.

## 4. Discussion

We hypothesized that the tandem administration of GnRH and PG on Days 0 and 7 of the 7 & 7 Synch protocol would reduce variation in luteal and follicular status on Day 14, increase the proportion of females expressing behavioral estrus prior to FTAI, and improve P/AI; however, the results observed suggest no meaningful improvements in these responses. In practice, ES is used in beef herds consisting of both cycling and acyclic females, due to variation in days postpartum, body condition, and nutritional status, as well as pubertal status in heifers [14,18,19]. We hypothesized that the incorporation of both PG and GnRH in the presynchronization portion of the 7 & 7 Synch protocol would improve control over ovarian dynamics among cows of varying cyclicity status. Minimizing variation among females in luteal status and follicular maturity results in more synchronous estrus and ovulation, leading to greater P/AI when using FTAI [20]. The results observed support the hypothesis that the inclusion of GnRH, as an ovulatory stimulus at the start of the 7 & 7 Synch treatment, can be performed without compromising the control obtained among cycling females; however, the overall pregnancy outcomes were not meaningfully improved for acyclic females.

The present results for CL status on Day 7 in the modified treatments indicate that a large proportion of females ovulated in response to the GnRH administered on Day 0. There was no significant interaction of pre-treatment luteal status and treatment, suggesting that a high ovulation rate was obtained even among acyclic cows. Although some reports in dairy cattle have indicated a reduced effect of both PG and GnRH when administered in tandem [21], Yousuf [22] reported that the administration of both PG and GnRH on the same day, seven days prior to the start of Ovsynch, was a simple and effective presynchronization strategy. For females ovulating in response to GnRH on Day 0 and subsequently recruiting a new follicular wave, the seven days between GnRH administrations should allow sufficient time for the maturation of an LH-responsive dominant follicle [23,24]. For females failing to ovulate in response to GnRH on Day 0, yet undergoing luteolysis in response to PG, the reduction in circulating P4 concentrations combined with the subluteal P4 from the CIDR should prevent atresia of the dominant follicle [6]; moreover, if the dominant follicle of such a female were already static or regressing, the seven-day interval before GnRH should provide adequate time for the maturation of a dominant follicle from the subsequent follicular wave [23]. Observations of a large proportion of cows presenting with CL on Day 14 in the 7 & 7 + G and even in the 7 & 7 + G + P treatments suggest a high ovulation rate in response to GnRH administered on Day 7 of the treatment schedule.

Across parities and pre-treatment luteal status classifications, treatment with GnRH on Day 0 resulted in a greater proportion of females presenting with CL on Day 7 when treated with the modified treatments (63%) when compared to females treated with 7 & 7 Synch (38%), in which luteolysis is induced intentionally for a large proportion of females. Because females treated with the standard 7 & 7 Synch protocol primarily have subluteal circulating concentrations of P4 levels over the following seven days, a greater frequency of LH release is anticipated to result in greater follicular size [25]. This may explain our observation of multiparous females treated with the modified treatments presenting with smaller Day 7 LFD (7 & 7 + G: 12.2 ± 0.20 mm; 7 & 7 + G + P: 12.4 ± 0.20 mm) compared to multiparous females treated with 7 & 7 Synch (13.1 ± 0.29 mm). It is worth noting, however, that mean follicle sizes in all treatments would generally be considered inducible to ovulate via GnRH administration (>10.5 mm) [26].

The inclusion of the 7 & 7 + G + P treatment in this experimental design allowed for the testing of hypotheses related to CL number on Day 14 and resulting potential effects on the expression of behavioral estrus and P/AI. While previous research reported no difference in P/AI between females that present with a single versus multiple CL at CIDR removal, females presenting with multiple CL have elevated circulating progesterone concentrations [27] and differ in the timing of onset of estrus [28]. Maximizing the proportion of females that synchronously express behavioral estrus prior to FTAI is a high research priority, as females expressing behavioral estrus prior to FTAI have significantly greater P/AI [29]. Although, in theory, the inclusion of an ovulatory stimulus on Days 0 and 7 increased the likelihood of multiple CL and, thus, greater circulating P4 concentrations in females treated with the 7 & 7 + G protocol, the proportion of females expressing estrus prior to FTAI and the pregnancy rates resulting from FTAI in this treatment suggest minimal cause for concern. It should also be noted that Day 14 LFD was similar across treatments, suggesting minimal impact on follicular maturity. Previous reports indicate the follicle sizes observed in all treatments would be capable of ovulating in response to GnRH administration [26,30].

The parity effects and interactions observed in this experiment highlight the greater likelihood of multiparous beef cows having favorable responses during estrus synchronization. Multiparous cows were more likely to be estrous cycling prior to the start of the protocol, were more likely to present with CL on Day 14 of the protocol, and were more likely to express behavioral estrus prior to FTAI. Across treatments and parities, females that expressed behavioral estrus prior to FTAI had greater P/AI (59%) when compared with females that failed to express estrus (35%). The proportion of multiparous females expressing behavioral estrus prior to FTAI was greatest among females treated with 7 & 7 + G + P (90%) when compared to those treated with 7 & 7 Synch (78%) or 7 & 7 + G (79%). Generally, a greater proportion of females expressing behavioral estrus prior to FTAI would be anticipated to result in greater P/AI, so long as the fertility of estrous females is not otherwise compromised (e.g., ovulation of a persistent follicle or poor timing of insemination for a subset of females). Thus, an increase in the proportion of females expressing estrus without an associated increase in P/AI raises questions. It is important to note that, among multiparous females treated with 7 & 7 + G + P, there was a lower proportion with CL on Day 14 (82%) when compared to those treated with 7 & 7 Synch (90%) or 7 & 7 + G (87%). Day 14 CL response in this experiment is of practical significance, as females failing to present with CL at CIDR removal are likely to vary with respect to timing of estrus onset in comparison to luteal females. Additionally, in theory, this subset of animals may potentially ovulate a persistent follicle [25]. Oocytes from persistent follicles have reduced fertility due to their extended exposure to an increased frequency of LH pulses, which causes the premature resumption of meiosis [25,31,32]. However, although a notable proportion of females on the 7 & 7 + G + P treatment presented with no luteal tissue on Day 14 (27%; 124/464), these females achieved P/AI comparable to that observed among females presenting with no luteal tissue on Day 14 in other treatments (7 & 7 Synch: 64% if estrous, 33% if non-estrous; 7 & 7 + G: 49% if estrous, 11% if non-estrous; 7 & 7 + G + P: 56% if estrous, 33% if non-estrous).

## 5. Conclusions

In conclusion, tandem administration of GnRH and PG at the start of the 7 & 7 Synch protocol was effective from an ovarian response standpoint, in that this presynchronization strategy resulted in convergent ovarian presentation among females with initially varying luteal status pretreatment. These hormones perform two distinct physiological functions and, thus, create potential for initially divergent ovarian responses (i.e., Day 7 ovarian presentation) among females administered both GnRH and PG in tandem at the start of the protocol. However, follicular maturity at the time of GnRH administration on Day 7 of the protocol clearly allowed for successful reconvergence of ovarian presentation by Day 14 of the protocol. However, incorporating the administration of GnRH in the presynchronization portion of the protocol failed to meaningfully enhance overall pregnancy rates to FTAI. The effects of incorporating PG administration in tandem with GnRH on Day 7 of the treatment schedule may require further investigation, as this resulted in an increased proportion of multiparous females expressing behavioral estrus prior to FTAI but with no accompanying increase in P/AI.

## Figures and Tables

**Table 1:** Number of cows and heifers by location, state, and season/year.

Location	State	Season/Year	N	
			Cows	Heifers
1	Missouri	Spring 2022	102	0
2	Missouri	Spring 2022	202	0
3	Missouri	Fall 2022	171	24
4	Missouri	Fall 2022	93	65
5	Missouri	Spring 2023	109	27
6	Missouri	Spring 2023	208	81
7	South Dakota	Spring 2023	140	0
8	South Dakota	Spring 2023	57	30
9	South Dakota	Spring 2023	259	78
10	Missouri	Fall 2023	136	27
11	Missouri	Fall 2023	72	21
12	Tennessee	Fall 2023	196	50
13	Missouri	Fall 2023	0	172

**Table 2:** Number of females and pre-treatment luteal status on Day 0 by treatment and parity.

Treatment ^1^	N	Pre-Treatment Luteal Status ^2^	
Parity		Proportion	%
7 & 7 Synch	765	465/765	61
Nulliparous	188	96/188	51
Primiparous	109	63/109	58
Multiparous	468	306/468	65
7 & 7 + G	769	468/769	61
Nulliparous	192	100/192	52
Primiparous	113	58/113	51
Multiparous	464	310/464	67
7 & 7 + G + P	786	480/786	61
Nulliparous	195	99/195	51
Primiparous	116	68/116	59
Multiparous	475	313/475	66

^1^ See Figure 1 for treatment descriptions. ^2^ Proportion of females that presented with corpora lutea (CL) at the start of the treatment schedule. Females were classified as luteal based on the observation of one or more CL, as assessed via transrectal ovarian ultrasonography.

**Table 3:** Day 7 and Day 14 corpora lutea (CL) status and largest follicle diameter (LFD) by treatment and parity.

Treatment ^1^	Day 7 LFD (mm)	Day 7 CL Status ^2^		Day 14 LFD (mm)	Day 14 CL Status ^2^	
Parity		Proportion	%		Proportion	%
7 & 7 Synch	12.1 ± 0.16	145/385	38 ^d^	11.3 ± 0.40	338/445	76
Nulliparous	10.2 ± 0.25 ^z^	27/131	21	11.1 ± 0.28	65/129	50
Primiparous	11.2 ± 0.54 ^xy^	28/49	57	11.7 ± 0.28	48/65	74 ^e^
Multiparous	13.1 ± 0.29 ^x^	90/205	44	11.7 ± 0.17	225/251	90 ^x^
7 & 7 + G	11.6 ± 0.22	247/390	63 ^c^	11.0 ± 0.38	346/450	77
Nulliparous	10.9 ± 0.18 ^z^	56/132	42	11.2 ± 0.22	81/133	61
Primiparous	11.5 ± 0.38 ^y^	38/51	75	11.9 ± 0.49	44/64	69 ^f^
Multiparous	12.2 ± 0.20 ^y^	153/207	74	11.3 ± 0.17	221/253	87 ^x^
7 & 7 + G + P	11.7 ± 0.22	262/415	63 ^c^	11.4 ± 0.44	338/462	73
Nulliparous	10.8 ± 0.18 ^z^	61/138	44	11.2 ± 0.25	76/134	57
Primiparous	12.5 ± 0.38 ^y^	35/54	65	12.6 ± 0.33	47/66	71 ^ef^
Multiparous	12.4 ± 0.20 ^y^	166/223	74	12.0 ± 0.19	215/262	82 ^y^

^1^ See Figure 1 for treatment descriptions. ^2^ Proportion of females luteal on Days 7 and 14. Females were classified as luteal based on the observation of one or more CL as assessed via transrectal ovarian ultrasonography. ^c,d,x,y,z^ Within the column, values with different superscripts differ (*p* < 0.05). ^e,f^ Within the column, values with different superscripts tended to differ (*p* = 0.08).

**Table 4:** Day 7 and Day 14 corpora lutea (CL) status and largest follicle diameter (LFD) by treatment and pre-treatment luteal status.

Treatment ^1^	Day 7 LFD (mm)	Day 7 CL Status ^2^		Day 14 LFD (mm)	Day 14 CL Status ^2^	
Pre-Treatment Luteal Status		Proportion	%		Proportion	%
7 & 7 Synch	12.1 ± 0.16	145/385	38 ^d^	11.3 ± 0.40	338/445	76
Luteal	12.7 ± 0.27	94/244	39	11.5 ± 0.47	220/281	78 ^a^
Non-luteal	11.3 ± 0.35	51/141	36	10.9 ± 0.45	118/164	72 ^b^
7 & 7 + G	11.6 ± 0.22	247/390	63 ^c^	11.0 ± 0.38	346/450	77
Luteal	11.9 ± 0.20	158/244	65	11.3 ± 0.42	220/274	80 ^a^
Non-luteal	11.2 ± 0.24	89/146	61	10.5 ± 0.43	126/176	72 ^b^
7 & 7 + G + P	11.7 ± 0.22	262/415	63 ^c^	11.4 ± 0.44	338/462	73
Luteal	12.1 ± 0.20	163/259	63	11.5 ± 0.53	232/290	80 ^a^
Non-luteal	11.2 ± 0.24	99/156	63	11.3 ± 0.53	108/174	62 ^b^

^1^ See Figure 1 for treatment descriptions. ^2^ Proportion of females luteal on Days 7 and 14. Females were classified as luteal based on the observation of one or more CL, as assessed via transrectal ovarian ultrasonography. ^a,b^ Within the column, values with different superscripts tended to differ (*p* = 0.09). ^c,d^ Within the column, values with different superscripts differ (*p* < 0.05).

**Table 5:** Proportion of females expressing behavioral estrus prior to fixed-time artificial insemination (FTA) by treatment and parity.

Treatment ^1^	N	Estrus Prior to FTAI ^2^	
Parity		Proportion	%
7 & 7 Synch	690	507/690	73
Nulliparous	179	110/179	61
Primiparous	96	72/96	75
Multiparous	415	325/415	78 ^a^
7 & 7 + G	701	526/701	75
Nulliparous	183	125/183	68
Primiparous	101	73/101	72
Multiparous	417	328/417	79 ^a^
7 & 7 + G + P	715	581/715	81
Nulliparous	187	120/187	64
Primiparous	106	81/106	76
Multiparous	422	380/422	90 ^b^

^1^ See Figure 1 for treatment descriptions. ^2^ Proportion of females expressing behavioral estrus prior to FTAI, based on females presenting with either a missing patch or ≥50% of the Estrotect patch coating being rubbed off. ^a,b^ Values with different superscripts differ (*p* < 0.05).

**Table 6:** Pregnancy rates to fixed-time artificial insemination (FTAI) by treatment and Day 14 corpora lutea (CL) status from the subset of females among which ovarian ultrasonography was performed on Day 14.

Treatment ^1^	N	Pregnancy Rate to FTAI ^3^	
CL Status Day 14 ^2^		Proportion	%
7 & 7 Synch	440	242/445	55
Luteal	335	184/335	55 ^a^
Non-luteal	105	58/105	55 ^a^
7 & 7 + G	446	254/446	57
Luteal	343	210/343	61 ^a^
Non-luteal	103	44/103	43 ^b^
7 & 7 + G + P	462	263/462	57
Luteal	339	198/339	58 ^a^
Non-luteal	123	65/123	53 ^ab^

^1^ See Figure 1 for treatment descriptions. ^2^ Proportion of females luteal on Day 14. Females were classified as luteal based on the observation of one or more CL, as assessed via transrectal ovarian ultrasonography. ^3^ Pregnancy rate to FTAI was determined via transrectal ultrasonography 60–100 days after FTAI. ^a,b^ Values with different superscripts differ (*p* < 0.05).

## Data Availability

The datasets generated from this experiment include information collected on producer operations and at multiple institutions. Data are not publicly available to protect the privacy of participants; however, partial or complete datasets may be available from the authors upon request.
